# Flow-controlled ventilation during EVLP improves oxygenation and preserves alveolar recruitment

**DOI:** 10.1186/s40635-020-00360-w

**Published:** 2020-11-25

**Authors:** Sofie Ordies, Michaela Orlitova, Tobias Heigl, Annelore Sacreas, Anke Van Herck, Janne Kaes, Berta Saez, Arno Vanstapel, Laurens Ceulemans, Bart M. Vanaudenaerde, Robin Vos, Johny Verschakelen, Geert M. Verleden, Stijn E. Verleden, Dirk E. Van Raemdonck, Arne P. Neyrinck

**Affiliations:** 1 Unit of Anesthesiology and Algology, Department of Cardiovascular Sciences, Katholieke Universiteit Leuven, Leuven, Belgium; 2 Department of Anesthesiology, University Hospitals Leuven, Herestraat 49, 3000 Leuven, Belgium; 3 Leuven Lung Transplant Group, Katholieke Universiteit Leuven, Leuven, Belgium; 4 Department of Thoracic Surgery, University Hospitals Leuven, Leuven, Belgium; 5 Laboratory of Respiratory Diseases and Thoracic Surgery (BREATHE), Department of Chronic Diseases, Metabolism and Ageing (CHROMETA), Katholieke Universiteit Leuven, Leuven, Belgium; 6 Department of Pneumology, University Hospitals Leuven, Leuven, Belgium; 7 Department of Pathology, University Hospitals Leuven, Leuven, Belgium; 8 Department of Radiology, University Hospitals Leuven, Leuven, Belgium

**Keywords:** Lung transplantation, Ex vivo lung perfusion, Flow-controlled ventilation, Donor management, Volume-controlled ventilation, Porcine large animal model

## Abstract

**Background:**

Ex vivo lung perfusion (EVLP) is a widespread accepted platform for preservation and evaluation of donor lungs prior to lung transplantation (LTx). Standard lungs are ventilated using volume-controlled ventilation (VCV). We investigated the effects of flow-controlled ventilation (FCV) in a large animal EVLP model.

Fourteen porcine lungs were mounted on EVLP after a warm ischemic interval of 2 h and randomized in two groups (*n* = 7/group). In VCV, 7 grafts were conventionally ventilated and in FCV, 7 grafts were ventilated by flow-controlled ventilation. EVLP physiologic parameters (compliance, pulmonary vascular resistance and oxygenation) were recorded hourly. After 6 h of EVLP, broncho-alveolar lavage (BAL) was performed and biopsies for wet-to-dry weight (W/D) ratio and histology were taken. The left lung was inflated, frozen in liquid nitrogen vapors and scanned with computed tomography (CT) to assess regional distribution of Hounsfield units (HU).

**Results:**

All lungs endured 6 h of EVLP. Oxygenation was better in FCV compared to VCV (*p* = 0.01) and the decrease in lung compliance was less in FCV (*p* = 0.03). W/D ratio, pathology and BAL samples did not differ between both groups (*p* = 0.16, *p* = 0.55 and *p* = 0.62). Overall, CT densities tended to be less pronounced in FCV (*p* = 0.05). Distribution of CT densities revealed a higher proportion of well-aerated lung parts in FCV compared to VCV (*p* = 0.01).

**Conclusions:**

FCV in pulmonary grafts mounted on EVLP is feasible and leads to improved oxygenation and alveolar recruitment. This ventilation strategy might prolong EVLP over time, with less risk for volutrauma and atelectrauma.

## Background

Lung transplantation (LTx) remains the last-resort solution for patients suffering from end-stage respiratory diseases [1]. However, the lack of suitable organs remains an important limiting factor for survival of wait listed candidates.

Ex vivo lung perfusion (EVLP) is a form of machine perfusion and has been introduced in the field of LTx to evaluate graft function outside the body [2]. During EVLP, the lungs are perfused with a normothermic solution and at the same time ventilated with positive pressure. Graft function is mainly monitored using standard physiological variables including compliance, pulmonary vascular resistance (PVR) and oxygenation. EVLP has the potential to assess organs physiologically and biologically prior to transplantation and facilitates longer preservation times compared to conventional hypothermic storage on ice [3]. In the future, active resuscitation of injured grafts by EVLP might be possible, but search of adequate therapeutic strategies is still ongoing [4].

To date, duration of EVLP is still limited up to 12 h clinically [5] and to 24 h in a large animal model [6–8]. A potential reason why EVLP is still limited in time might be the technology itself. The concept of EVLP is based on a physiological environment and assessment. However, the ex situ context cannot be completely compared to in vivo physiology. First, the graft is mounted in a static supine position in the organ chamber. We have demonstrated that prone position alters region distribution of edema accumulation [9]. Second, the graft is mechanically ventilated with positive airway pressure. Patients who receive mechanical ventilation with increased pressures (barotrauma), large volumes (volutrauma), repetitive alveolar opening and closing (atelectrauma) and asynchronous breathing (self-inflicting lung injury) are at risk for developing ventilator-induced lung injury (VILI), which leads to inferior outcome [10–12]. Therefore, one can presume that similar mechanisms of VILI might be important during ex vivo lung perfusion.

The two most frequently used ventilation modes during EVLP are volume-controlled ventilation (VCV) and pressure-controlled ventilation (PCV) [4]. In addition, it is not clear what would be the optimal recruitment maneuver to actively open collapsed alveoli, especially with absence of chest wall. These ventilation strategies only control the inspiratory and not the expiratory phase of the respiratory cycle. Expiration depends on passive elastic recoil of the lungs.

**Fig. 1:**
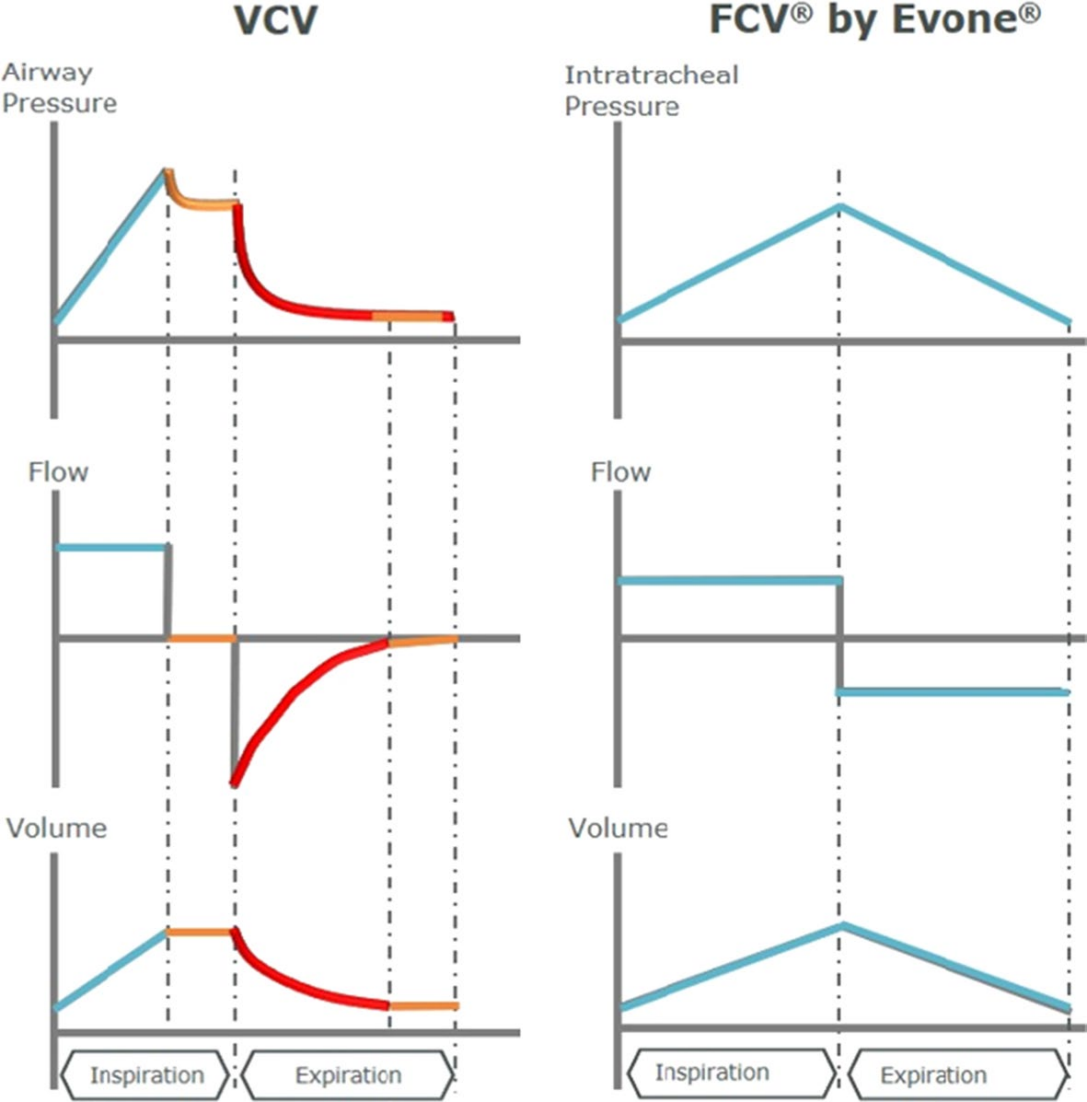
Volume-controlled ventilation (VCV) and flow-controlled ventilation (FCV). Visualization of the airway/tracheal pressures, flow and volume during conventional volume-controlled ventilation (VCV, left) and flow-controlled ventilation (FCV, right) during inspiration and expiration

Flow-controlled ventilation (FCV) is an innovative ventilation mode providing a constant flow during both inspiration and expiration, resulting in a linear increase and decrease in intratracheal and thus intrapulmonary pressure. FCV has been shown to enhance lung aeration, to prevent atelectasis and to reduce lung damage [13]. The airway/intratracheal pressure, gas flow and tidal volume patterns of VCV and FCV ventilation modes are illustrated in Fig. 1.

The effects of FCV ventilation have not been previously investigated in a large animal EVLP model. Therefore, the aim of this study was to explore effects of FCV during EVLP compared to conventional VCV. We hypothesized that FCV could improve alveolar recruitment and reduce VILI during EVLP compared to VCV.

## Methods

### Animals

Fourteen specific pathogen-free domestic male pigs (Topig20, Tojapigs, Escharen, The Netherlands) (median weight 39 kg (36.5–39.6 IQR), *n* = 7/group) were included in this study after approval by the Ethics Committee (P145/2016). Each animal received human care conform the “Principles of Laboratory Animal Care”, formulated by the National Society for Medical Research and “Guide for the Care and Use of Laboratory Animals,” prepared by the Institute of Laboratory Animal Resources and reported by the National Institutes of Health, USA (NIH Publication No. 86-23, revised 1996).

### Injury model and EVLP setup

Animals were sedated and anaesthetized as previously described [9]. Baseline venous and arterial blood samples were taken at 100% fraction of inspiratory oxygen (FiO_2_). Baseline respiratory and hemodynamic parameters were recorded with ICM^+®^ (Cambridge Enterprise, Cambridge, UK). Donor animals were ventilated with volume-controlled ventilation (VCV) 8 mL/kg, an inspiratory time-to-expiratory ratio (I:E) of 1:2 and a positive end-expiratory pressure (PEEP) of 5 cm H_2_O, and respiratory rate (RR) was dependent on maintenance of expiratory carbon dioxide (CO_2_) levels between 40 and 45 mmHg. Median sternotomy was performed and heparin (300 U/kg) (LEO Pharma BV, Amsterdam, The Netherlands) was administered prior to introduction of cardiac fibrillation (10 V until witnessed cardiac arrest).

After cardiac arrest, lungs were left untouched and deflated in situ for 2 h of warm ischemia. Sequentially, the grafts were retrogradely flushed with 1.5 L OCS™ solution (Transmedics, Andover, MA, USA). The first 0.5 L flush solution was administered at room temperature (21 °C), the next 1 L was at 4 °C. The lungs were inflated, stored on ice and prepared for EVLP. Our EVLP setup was as follows: acellular perfusate, closed atrium and flow calculated as 40% of cardiac output. A centrifugal pump, heat exchanger, gas exchanger, and reservoir enabled normothermic (37 °C), double-lung EVLP during 6 h as previously reported [9]. Briefly, the lungs were cannulated with XVIVO™ Lung Cannula Set (XVIVO Perfusion™, Göteborg, Sweden).

### Study groups and ventilation strategy

Lungs were randomized in two groups (*n* = 7/group). In the VCV group, donor lungs were ventilated according to standard VCV during EVLP (tidal volume (TV) of 7 mL/kg; I:E 1:2, 5 cm H_2_O PEEP and RR of 7 breaths/min) (Aestiva 3000; GE Healthcare Europe GmbH, Little Chalfont, UK) and an endotracheal tube (ETT) of 8.0 mm. A recruitment maneuver based on an increase of PEEP to 10 cm H_2_O and inspiratory pressure to 25 cm H_2_O during 60 s was applied.

In the FCV group, grafts were ventilated with FCV (Evone^®^, Ventinova Medical, Eindhoven, The Netherlands) during EVLP. The settings of FCV are based on an adjustment of the gas flow, PEEP and inspiratory pressure only. Ventilatory rate and volume cannot be independently set and are dependent on the driving pressure (inspiratory pressure–PEEP) and flow. For these experiments, the driving pressure was set to reach a TV of 7 mL/kg and the flow adjusted to reach a RR of 7 breaths/min. I:E was 1:1 and 5 cm H_2_O PEEP was maintained during 6 h of EVLP. A special requirement to control the gas flow, is the use of a narrow-bore ETT with direct measurement of intratracheal pressure (Tritube^®^; Ventinova Medical, Eindhoven, The Netherlands). In both groups, FiO_2_ during EVLP was 21%. Hourly blood gases were taken with a FiO_2_ of 100%.

### Evaluation of graft function and lung injury

All lungs remained on EVLP for 6 h. During EVLP, hemodynamic [pulmonary artery pressure (PAP), left atrial pressure (LAP)] and respiratory parameters (compliance, RR, flow) were monitored. Since compliance was calculated differently, based on extra-tracheal airway pressure proximally of the ETT in VCV and intratracheal pressure in FCV, static compliance was recalculated in the VCV group using following formula expiratory TV/(P_plateau_–PEEP). For FCV, dynamic compliance calculated by Evone^®^ was based on inspiratory tidal volume measured by mass flowmetry and driving pressure. Delta (Δ) of PVR and oxygenation and compliance were calculated as difference of the respective parameter between 6 and 1 h of EVLP.

At the end of EVLP, a ventral and dorsal biopsy of the right lung were taken for pathology and for calculation of wet-to-dry weight (W/D) ratio. Biopsies were processed as previously reported [9], hematoxylin and eosin staining was scored by a pathologist blinded for group information. A composite score for pathology was used, including congestion, interstitial thickening and alveolar edema [each parameter scored between 0 (absent) and 3 (manifest present)]. The presence of neutrophils was noted as 0 (absent) or 1 (present). The sum of all scores was calculated and divided by the maximum score 20 to illustrate the percentage of injury. Lungs were weighed prior to and after EVLP. Samples for W/D were mounted in a heated oven of 80 °C and weighed after 72 h. After EVLP, a broncho-alveolar lavage (BAL) sample was taken in the right middle lobe by applying two times 30 mL of saline 0.9%. Pooled fractions were obtained and a 100µL cytospin was stained with Diff-Quick (Dade Behring, Newark, NJ, USA) to calculate total and differential cell counts [14]. Finally, the left lung was inflated at a constant inspiratory pressure of 25 cm H_2_O and frozen into liquid nitrogen fumes and computed tomography (CT) scanned (Siemens Somatom scanner, Erlangen, Germany) at 120 kV and 110 mAs. HOROS^®^ [DICOM Viewer, Version 3 (GPL-3.0)] was used to calculate CT density measurements of the left lungs. Regional CT Hounsfield units (HU) were acquired by Fiji (ImageJ 1.52p, National Institutes of Health, USA, http://image.nih.gov/ij) by manually creating a mask for every lung to remove background noise and sequentially using the analysis ‘Histogram’ to reveal the distribution of HU over the grafts. HU were considered as follows: portions between − 1000 and − 900 HU as ‘over-inflated’, between − 900 to − 500 as ‘well-aerated’, − 500 and − 100 HU ‘poorly aerated’ and − 100 and 0 HU ‘not aerated’ [15].

### Statistics

Data were visualized as median [25–75% interquartile range (IQR)] unless otherwise stated. Since all data were not normality distributed, non-parametric tests were applied. Mann–Whitney test was used to compare both groups and Friedman test for repeated measurements (Sidak test for post hoc analysis). All analyses were performed with Graphpad^®^ Prism 7.04 (GraphPad Software Inc, La Jolla, CA, USA). *p* values less than 0.05 were considered significant.

## Results

### Baseline characteristics

**Fig. 2:**
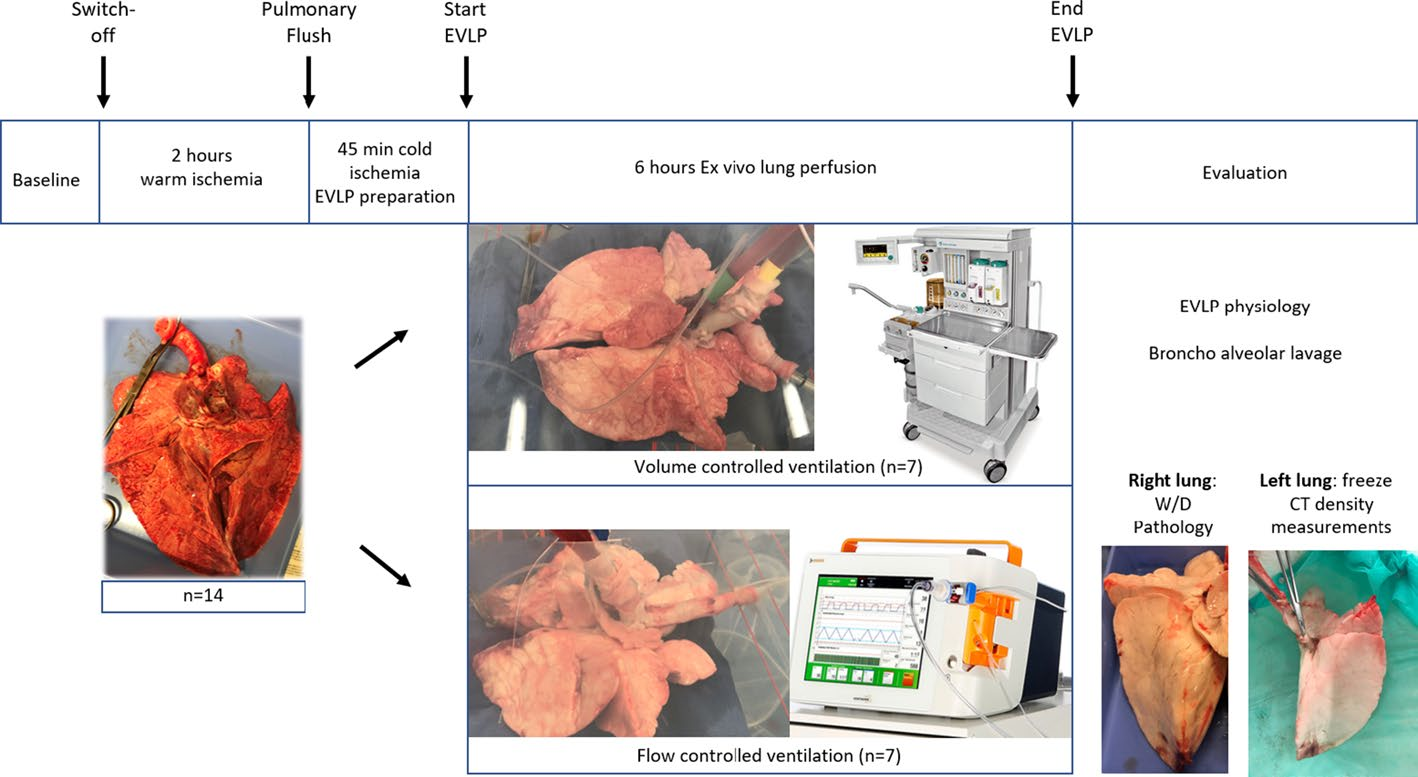
Study design

Baseline characteristics are listed in Table 1 and did not differ between both groups. Study design is illustrated in Fig. 2.

**Table 1:** Baseline animal characteristics, ischemic times and perfusate composition

Baseline animal characteristics	VCV (*n* = 7) Median (25–75% IQR)	FCV (*n* = 7) Median (25–75% IQR)	*p* value
Pig weight (kg)	38.8 (36.3–39.6)	39.6 (36.0–41.4)	0.40
Lung compliance donor (mL/cm H_2_O)	34 (32–36)	36 (28–38)	0.99
PaO_2_/F_i_O_2_ ratio at 100 F_i_O_2_ (mmHg)	529 (469–552)	546 (523–581)	0.21
Respiratory rate (breaths/min)	22 (20–22)	20 (20–22)	0.59
MAP (mmHg)	110 (99–112)	106 (90–107)	0.43
Heart rate (bpm)	108 (89–116)	84 (72–106)	0.32
Hb (mg/dL)	10.9 (10.6–11.1)	10.4 (10.2–10.5)	0.09
WBC count (10^9^/L)	14.6 (13.2–17.2)	14.8 (11.1–16.4)	0.62
Neutrophils (%)	40 (31–41)	31 (27–33)	0.18
Ischemic time			
Warm ischemic time (min)	120 (120–120)	120 (116–120)	0.27
Cold ischemic time (min)	44 (43–48)	48 (47–50)	0.13
Perfusate composition			
Glucosis (mg/dL)	234 (230–239)	229 (222–234)	0.18
HCO_3_^−^ (mmol/L)	32.5 (31.2–34.8)	32.5 (31.5–32.8)	0.98
K^+^ (mmol/L)	3.5 (3.5–3.6)	3.5 (3.5–3.6)	0.88
Na^2+^ (mmol/L)	158 (158–159)	156 (155–158)	0.09
Cl^−^ (mmol/L)	106 (105–107)	105 (104–107)	0.71
Ca^2+^ (mmol/L)	0.51 (0.49–0.54)	0.48 (0.42–0.51)	0.10
Osmolality (mmol/kg H_2_O)	318 (317–321)	319 (314–320)	0.92
Albumin (g/L)	72 (69–75)	71 (70–73)	> 0.99

Baseline animal characteristics, ischemic times and perfusate composition did not differ between VCV and FCV

*VCV* volume-controlled ventilation, *FCV* flow-controlled ventilation, *IQR* interquartile range, *kg* kilogram, *mL* milliliters, *P*_*a*_*O*_*2*_ partial pressure of oxygen, *F*_*i*_*O*_*2*_ fraction of inspiratory oxygen, *cm* centimeter, *bpm* beats per minute, *Hb* hemoglobin, *mg* milligram, *dL* deciliter, *WBC* white blood cell, *min* minutes, *mmol* millimole, *L* liters, *g* grams

### EVLP physiology

Inspiratory flow of VCV was 60 L/min, median inspiratory flow of FCV was 3 (3–4) L/min. Perfusate flows were not different between both groups VCV 1.51 (1.51–1.52) L/min vs. FCV 1.50 (1.49–1.51) L/min (*p* = 0.77). The median delivered tidal volumes were 269 (252–278) mL in VCV, and 271 (249–286) mL in FCV (*p* > 0.99). Median PAP and median LAP were similar between both groups (VCV: 14.3 (13.6–14.7) mmHg vs. FCV: 15.6 (14.7–16.0) mmHg; *p* = 0.21; and VCV: 3.2 (2.9–3.5) mmHg vs. FCV: 3.3 (2.9–3.5) mmHg; *p* = 0.87), respectively).

**Fig. 3:**
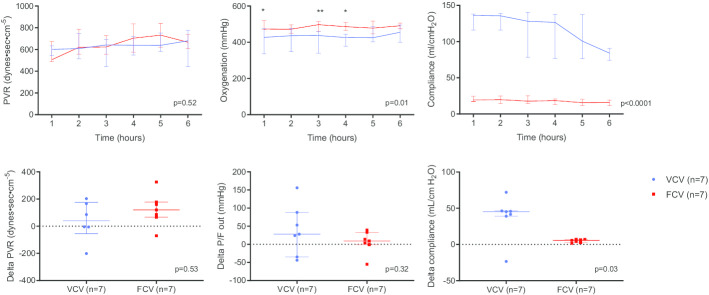
EVLP physiology. EVLP physiology is visualized. Pulmonary vascular resistance did not differ between VCV and FCV (*p* = 0.52) (left, upper panel). Oxygenation, PaO_2_/F_i_O_2_ ratio was significantly lower in VCV compared to FCV (*p* = 0.01) (middle, upper panel). Post hoc analysis revealed differences at 1 h, 3 h and 4 h (*p* = 0.04, *p* = 0.007 and *p* < 0.05, respectively). The decrease in lung compliance was significantly higher in VCV compared to FCV (*p* = 0.03) (right, upper panel). There were no differences (Δ) of PVR and oxygenation between 6 and 1 h of EVLP between both groups (*p* = 0.53, *p* = 0.32) (left and middle, lower panel). The Δ compliance was significantly higher in VCV compared to FCV (*p* = 0.03) (right, lower panel)

Results of the 6-h EVLP interval are visualized in Table 2 and Fig. 3. PVR was comparable between VCV and FCV (*p* = 0.52). Oxygenation ratio (PaO_2_/F_i_O_2_ (P/F) ratio) was significantly higher in FCV compared to VCV (*p* = 0.01). Sidak post hoc analysis revealed significant differences at time points 1, 3 and 4 h during EVLP (*p* = 0.04, *p* < 0.01 and *p* < 0.05, respectively). Because of the difference in measurement of compliance, no conclusions can be withdrawn comparing the absolute values between both groups. In addition to the absolute values, we analyzed the difference (Δ) of each physiological parameter between 6 and 1 h of EVLP. No differences were detected in ΔPVR (*p* = 0.53) and Δ oxygenation (*p* = 0.32). The Δ compliance between 6 and 1 h of EVLP was significantly greater in VCV compared to FCV (*p* = 0.03).

**Table 2:** Peak, plateau pressure and perfusate lactate, pCO_2_ and pH values between both groups

	VCV (*n* = 7) median with IQR	FCV (*n* = 7) median with IQR	*p* value (Mann–Whitney)
Peak pressures at 6 h EVLP	20 (17–25) cm H_2_O	22 (20–27) cm H_2_O	*p* = 0.51
Plateau pressures at 6 h EVLP	8 (8–9) cm H_2_O	–	–
Intratracheal pressures at 6 h EVLP	–	22 (20–27) cm H_2_O	–
pCO_2_ at 6 h EVLP ‘inflow’ (pulmonary artery)	42 (40–43) mmHg	41 (39–44) mmHg	*p* > 0.99
pCO_2_ at 6 h EVLP ‘outflow’ (left atrium)	36 (35–39) mmHg	37 (36–39) mmHg	*p* = 0.38
pH at 6 h EVLP outflow	7.40 (7.39–7.41)	7.38 (7.37–7.40)	*p* = 0.23
Lactate at 6 h EVLP outflow	12 (11–13) mg/dL	13 (13–14) mg/dL	*p* = 0.18

*VCV* volume-controlled ventilation, *FCV* flow-controlled ventilation, *IQR* interquartile range

Weight gain between pre- and post-EVLP was comparable between both groups [VCV 195 g (− 27 to 230) and FCV 37 g (− 53 to 130); *p* = 0.38].

### Broncho-alveolar lavage: total and differential cell count

Analyses of the BAL fluid did not reveal any difference in median total cell count (× 10^9^ cells/mL) (2.9 (2.7–3.0) in VCV vs. 3.0 (2.5–3.4) in FCV; *p* = 0.97) or in differential cell count: number of macrophages (90% (88–94) in VCV vs. 90% (87–91) in FCV; *p* = 0.62) and neutrophils [10% (6–12) in VCV vs. 10% (9–13) in FCV; *p* = 0.62] were similar. Cell viability was comparable between both groups [38% (25–40) in VCV vs. 46% (36–66) in FCV; *p* = 0.26].

### W/D ratio, histology and CT analyses

**Fig. 4:**
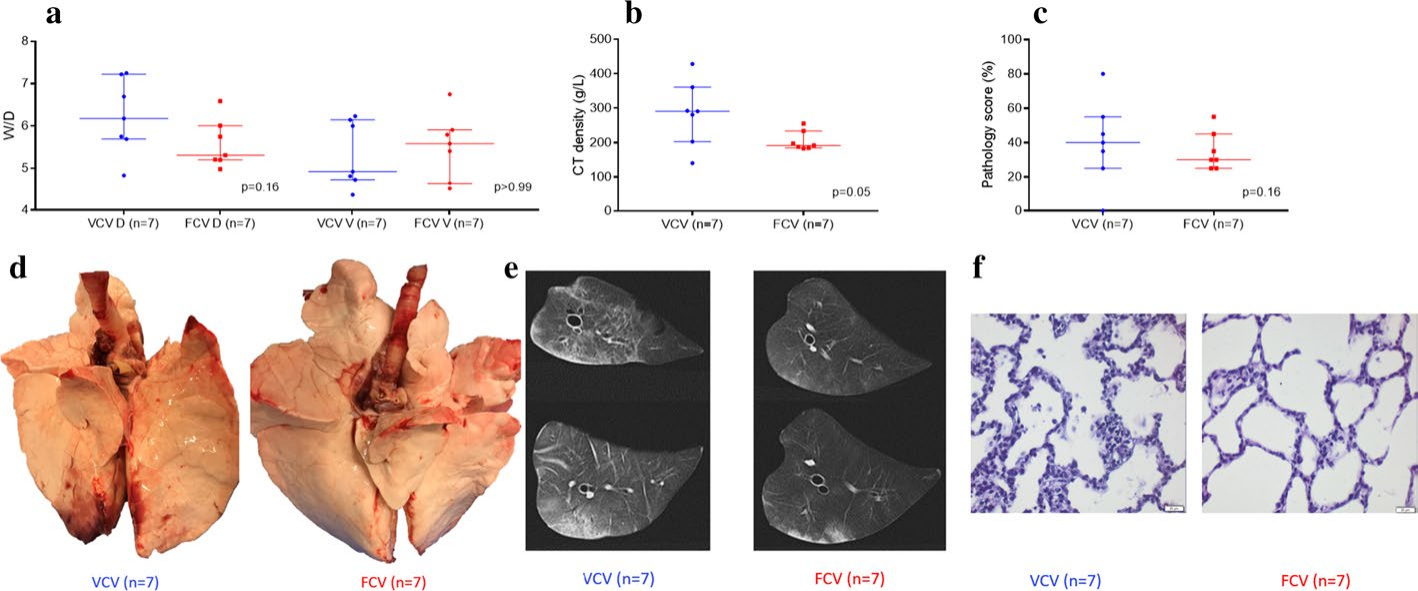
W/D ratio, CT density measurements and pathology. Wet-to-dry (W/D) weight ratios were similar between VCV and FCV (*p* = 0.16) (left, upper panel). CT density measurements tended to be higher in VCV compared to FCV (*p* = 0.05) (middle, upper panel). Pathology composite score (congestion, interstitial thickening, neutrophil influx and alveolar edema, in %) did not differ between both groups (*p* = 0.16) (right upper panel). Representative macroscopic pictures of grafts after 6 h of EVLP (left, lower panel), representative CT-graphic images (middle, lower panel) and representative histological images (right, lower panel) of VCV and FCV are demonstrated

**Fig. 5:**
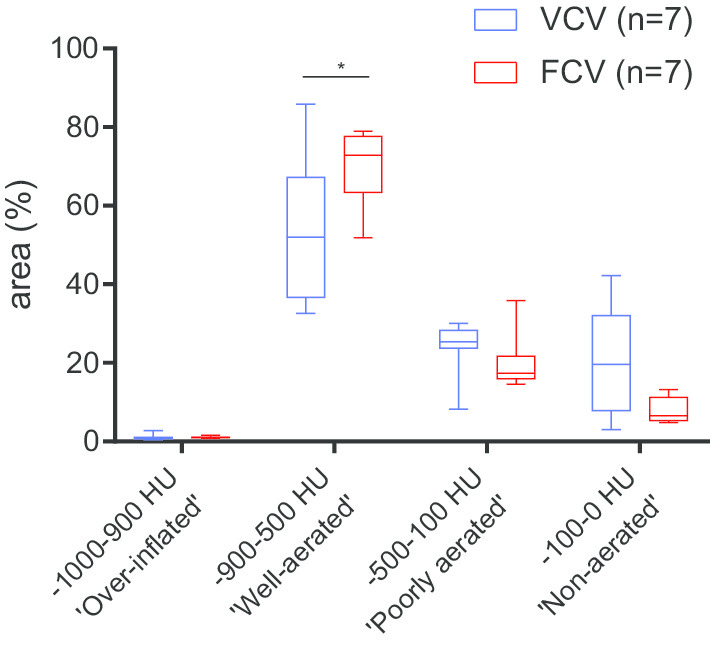
Regional distribution of Hounsfield units. The distribution of proportion of Hounsfield units (HU) is visualized in this figure. The proportion of following HU intervals was not different between VCV and FCV: − 1000 and − 900 HU (*p* > 0.99) and between − 500 and − 100 (*p* = 0.94). The proportion of − 900 and − 500 HU was increased in FCV compared to VCV (*p* = 0.01) and the proportion of interval -100 and 0 HU tended to be lower in FCV compared to VCV (*p* = 0.06)

Results of W/D ratio, CT imaging and pathology are visualized in Fig. 4. Macroscopic aspects of the grafts after EVLP are illustrated in Fig. 4d. Representative CT images of both groups are visualized in Fig. 4e and pathology images are shown in Fig. 4f. In line herewith is the absence of difference in W/D ratios between groups (*p* = 0.16). Total CT density measurements tended to be lower in FCV (*p* = 0.05; Fig. 4e). There was no difference in composite pathology score (*p* = 0.16; Table 3). Further analyses of HU distribution throughout the lungs are illustrated in Fig. 5. No differences were observed in the proportion of − 1000 and − 900 HU (‘over-inflated’) when comparing VCV and FCV (*p* > 0.99), nor between − 500 and − 100 HU (‘poorly aerated’ *p* = 0.94). A higher proportion of HU between − 900 and − 500 was observed in FCV compared to VCV (‘well-aerated’, *p* = 0.01). The proportion between − 100 and 0 HU tended to be lower in FCV compared to VCV (‘non-aerated’ *p* = 0.06).

**Table 3:**
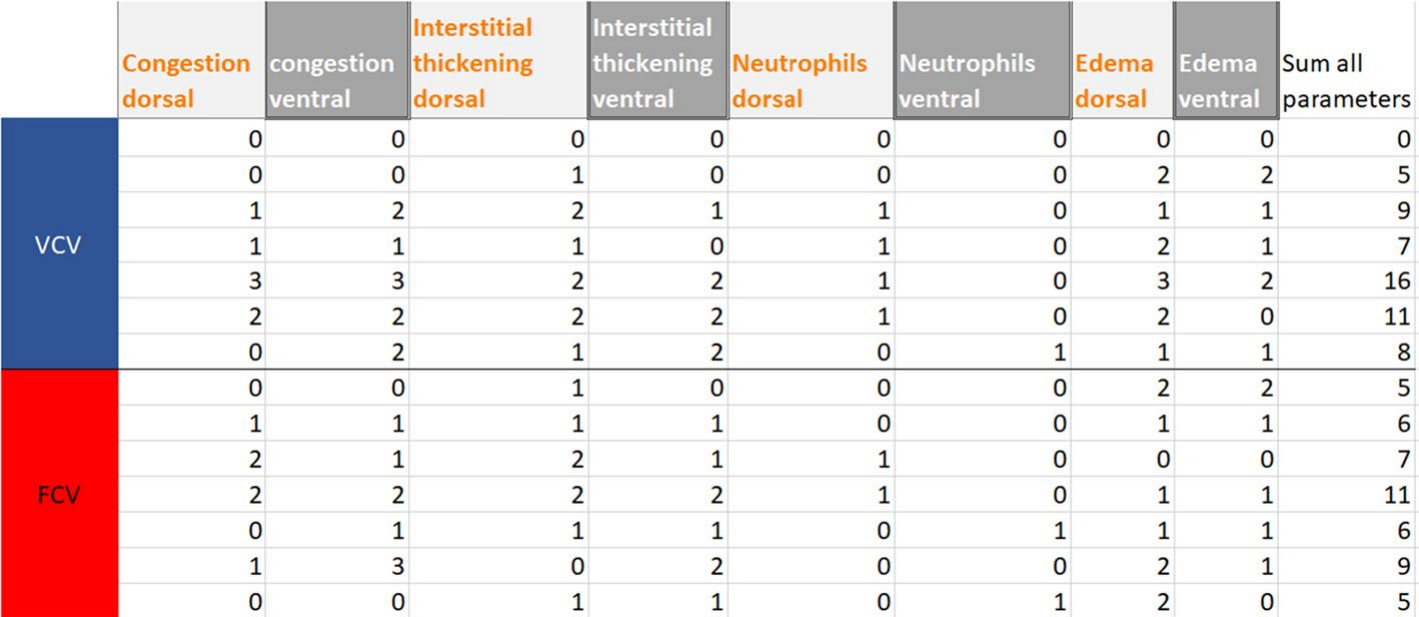
Histology scores

Every graft was scored for congestion (0–3), interstitial thickening (0–3), presence of neutrophils (0–1) and edema (0–3) for every dorsal and ventral area. Data are shown of both groups: VCV (*n* = 7, upper part, blue) and FCV (*n* = 7, lower part, red)

*VCV D* dorsal area of volume-controlled group, *VCV V* ventral area of volume-controlled group, *FCV D* dorsal area of flow-controlled group, *FCV V* ventral area of flow-controlled group

## Discussion

This is the first study evaluating the effects of the novel ventilation method FCV in a large animal EVLP model. We demonstrated that FCV as ventilation strategy during EVLP is feasible and might improve graft function. When comparing FCV and VCV, oxygenation was superior in FCV and with a better maintenance of recalculated compliance over 6 h in FCV. In addition, the proportion ‘well-aerated’ lung parts (HU between -900 and -500) was higher in FCV. These parameters suggest an improved preservation of alveolar recruitment in FCV compared to conventional VCV. Further studies with longer EVLP times and transplantation models are necessary to confirm that FCV better preserves lung grafts and limits injury during EVLP preservation.

However, in this study, there was no difference in total lung injury. Extravascular water content, measured by W/D ratios, weight gain after EVLP and cellular differentiation of BAL samples did not differ between FCV and VCV. Furthermore, pathology scores were similar between both groups. These findings suggest that in these 6 h of EVLP, FCV did not affect extravascular water accumulation nor inflammatory responses.

The main difference between FCV and VCV is that in FCV, both inspiration and expiration phase are controlled, whereas in VCV, expiration occurs by elastic recoil of the lungs [16]. The constant flow during expiration in FCV, might prevent sudden alveolar collapse and might delay complete emptying of the alveoli resulting in improved recruitment (less atelectasis) and better oxygenation [13]. The reduced number of collapsed alveoli decreased the risk for local overdistention alveoli (less volutrauma), and thereby potentially decreases an important driver of VILI.

Since atelectrauma and volutrauma both trigger inflammatory responses and contribute synergistically to VILI [10], development of new ventilation strategies which avoid these traumatic events are crucial—especially in donor lungs which are already at risk of developing ischemia–reperfusion lesions because of donor-related injury, emerging during ventilation after brain insult and further during procurement and preservation on ice.

The potential lung protective effects of controlling the expiratory flow were investigated by Goebel et al. [16]. Expiratory flow was slowed down by applying a flow restrainer in the expiratory limb of the circuit of a conventional ventilator and this flow-controlled expiration (so called “FLEX”) was evaluated in porcine ARDS lungs. Improved lung compliance and reduced lung pathology in the “FLEX” group compared to the conventionally ventilated group could be demonstrated. In addition, in healthy and injured pig lungs, “FLEX” was shown to lead to a more homogenous ventilation and shifting ventilation from ventral to dorsal parts [17]. Similar results were observed in healthy subjects [18].

FCV is a more sophisticated way to control the expiratory flow, as (in contrast to “FLEX”) it is linearized by actively regulating the egress of gas by controlled suctioning instead of using a passive flow restrainer. This allows to achieve a preferable I:E ratio of 1:1 without the risk of undesired auto-PEEP to build up [13]. Furthermore, the FCV ventilator has been designed and built to also meet the requirements of a measurement tool allowing to study dynamic lung mechanics by the combination of minimized technical dead space, precise volumetry (by mass flowmetry) and continuous intratracheal pressure measurement. However, in our study, these features were not applied to individualize the settings.

To our knowledge, this is the first study that uses the innovative FCV mode in an ex vivo setting. Other groups investigated the effects of FCV in vivo. Two previous studies [13, 19] compared the effects of FCV versus VCV in healthy pigs or ARDS pigs. Both studies demonstrated a significantly higher PaO_2_ during FCV at a lower minute volume.

Additionally, CT scans of FCV lungs revealed an increased proportion of normally aerated lung volume and a reduced area of poorly or non-aerated lung parts compared to VCV [13, 19], which is in line with the observations in our study. More recently, a randomized controlled trial was published, comparing FCV with VCV in patients undergoing laryngeal surgery [20]. FCV resulted in improved lung aeration and increased respiratory system compliance, while using a lower inspiratory plateau pressure compared to VCV.

Next to volutrauma, barotrauma and atelectrauma, mechanical power, has become acknowledged as a causal factor for development of VILI [21]. Excess of mechanical power applied to lungs has been shown to have deleterious effects [21, 22].

As explained, FCV is based on generating a constant, linearized low flow into and out of the lungs, resulting in slow, continuous, linear increases and decreases of intratracheal and thus intrapulmonary pressures that are just enough to establish mechanical ventilation with efficient gas exchange. The sudden alveolar pressure drop during uncontrolled passive expiration in conventional ventilation is prevented. In other words, the amount of energy generated by the FCV ventilator is just enough to establish sufficient gas exchange. Thereby, the impact on the lung tissue by dissipated energy is kept to a minimum, enabling ventilation with a markedly reduced risk of lung damage. Recently, clear theoretical evidence was provided for lower energy dissipation in the lungs by FCV as compared to VCV or PCV [23]. This theory was further validated in a patient, showing that the energy dissipation was just 0.17 J/L, which is even lower than values reported for spontaneous breathing (0.2–0.7 J/L) [24]. In the current study, dissipated energy during VCV and FCV was not calculated, but the described lower energy dissipation by FCV may have contributed to the observed positive effects.

Limitations of this study might have been the large animal model with 2 h of warm ischemic time, followed by 6 h of EVLP. One could argue that this is limited in time, but physiologic differences already became visible during this ventilation strategy after 1 h (increased oxygenation in FCV). This increase in oxygenation might be contributed by increased P_trach_ in FCV. Compliance was recalculated differently between both groups and therefore complicates true comparison. Therefore, we reported the decline of compliance in time within each group as an important parameter of onset of lung injury. We did not record and compare mean AWP between both groups. In this study, we applied standard settings of each ventilation mode. We cannot exclude a higher mean AWP in FCV (also because of differences in I:E ratio) which might have contributed to our findings. Further research should clarify the influence of mean AWP between both ventilation modes before generalization of these results. In addition, regional CT-graphic changes were already visible after 6 h of EVLP. These scans were taken at the end of every experiment. Whether a prolonged period of EVLP may have revealed differences in total lung injury remains to be studied. Though, the improved alveolar recruitment and maintenance of lung mechanics (compliance) indicate that FCV could lead to less VILI and enable longer perfusion times.

## Conclusions

In conclusion, we observed that FCV leads to increased aerated regions in the lung during EVLP compared to conventional VCV in a large animal model. Future studies are necessary to confirm that FCV better preserves lung grafts and limits injury during EVLP preservation. This opens the perspective of a possible extension of EVLP duration and better preservation of graft quality during EVLP.

## Data Availability

The dataset used and analyzed during the current study is available from the corresponding author on reasonable request.

## Abbreviations

BAL: Broncho-alveolar lavage
CO_2_: Carbon dioxide
CT: Computed tomography
EVLP: Ex vivo lung perfusion
FCV: Flow-controlled ventilation
FiO_2_: Fraction of inspired oxygen
HU: Hounsfield unit
I:E: Inspiratory time-to-expiratory ratio
LAP: Left atrial pressure
LTx: Lung transplantation
P/F: PaO_2_/F_i_O_2_
PAP: Pulmonary artery pressure
PCV: Pressure-controlled ventilation
PEEP: Positive end-expiratory pressure
PVR: Pulmonary vascular resistance
RR: Respiratory rate
TV: Tidal volume
VCV: Volume-controlled ventilation
VILI: Ventilator-induced lung injury
W/D ratio: Wet-to-dry weight ratio
